# Effects of Rapid Recovery on Alcohol Hangover Severity: A Double-Blind, Placebo-Controlled, Randomized, Balanced Crossover Trial

**DOI:** 10.3390/jcm9072175

**Published:** 2020-07-09

**Authors:** Andrew Scholey, Elizabeth Ayre, Ann-Kathrin Stock, Joris C Verster, Sarah Benson

**Affiliations:** 1 Centre for Human Psychopharmacology, Swinburne University, Melbourne, VIC 3122, Australia; andrew@scholeylab.com (A.S.); besayre24@gmail.com (E.A.); j.c.verster@uu.nl (J.C.V.); 2 Cognitive Neurophysiology, Department of Child and Adolescent Psychiatry, Faculty of Medicine, TU Dresden, Fetscherstr. 74, 01307 Dresden, Germany; Ann-Kathrin.Stock@uniklinikum-dresden.de; 3 Division of Pharmacology, Utrecht Institute for Pharmaceutical Sciences (UIPS), Utrecht University, 3584CG Utrecht, The Netherlands

**Keywords:** hangover, alcohol, hangover treatment, inflammation, liver function

## Abstract

The aim of this study was to evaluate the efficacy of putative hangover treatment, Rapid Recovery, in mitigating alcohol hangover (AH) symptom severity. Using a double-blind, randomized, placebo-controlled, balanced crossover design, 20 participants attended the laboratory for two evenings of alcohol consumption, each followed by morning assessments of AH severity. Participants were administered Rapid Recovery and placebo on separate visits. In the first testing visit, participants self-administered alcoholic beverages of their choice, to a maximum of 1.3 g/kg alcohol. Drinking patterns were recorded and replicated in the second evening testing visit. In the morning visits, AH severity was assessed using questionnaires measuring AH symptom severity and sleep quality, computerized assessments of cognitive functioning as well as levels of blood biomarkers of liver function (gamma-glutamyl transferase (GGT)) and inflammation (high-sensitive C-reactive protein (hs-CRP)). There were no differences in the blood alcohol concentrations (BAC) obtained in the Rapid Recovery (mean = 0.096%) and placebo (mean = 0.097%) conditions. Participants reported significantly greater sleep problems in the Rapid Recovery compared to placebo condition, although this difference was no longer significant following Bonferroni’s correction. There were no other significant differences between Rapid Recovery and placebo. These data suggest that Rapid Recovery has no significant effect on alcohol hangover nor on associated biomarkers.

## 1. Introduction

Alcohol hangover (AH) is defined as the combination of negative mental and physical symptoms which can be experienced after a single episode of alcohol consumption, starting when blood alcohol concentration (BAC) approaches zero [1,2]. It is characterized by a general state of malaise and a range of physical and psychological symptoms including headache [3,4], fatigue [5], nausea [4] and reduced cognitive functioning [6,7,8]. These symptoms negatively impact daily activities such as driving [9,10], job performance [11,12] and studying [4].

AH is pervasive, affecting 75% of all social drinkers [13]. As well as subjective effects it contributes to significant economic costs. It is estimated that, due to associated absenteeism and presenteeism, AH costs the UK economy between £1.2 billion and £1.4 billion per year [14] (i.e., approximately US $1.5 to $1.7 billion) and the Australian economy over AUS $3 billion annually [15] (i.e., approximately US $1.8 billion). Assessing the full cost, beyond absenteeism and presenteeism, it has been estimated that AH costs the American economy some US $179 billion per year [16].

The physiological causes of AH are largely unknown. Analyses of blood, saliva and urine samples indicate that concentrations of various hormones, electrolytes, free fatty acids, triglycerides, lactate, ketone bodies, cortisol, glucose and biomarkers of dehydration do not appear to correlate with hangover symptom severity [17,18]. In a recent review of biological factors that contribute to AH, Palmer et al. [19] concluded that alcohol metabolites, inflammatory factors, neurotransmitter alterations and mitochondrial dysfunction are the most likely contributors to AH severity.

Alcohol is predominately broken down in the liver, where it is metabolized by alcohol dehydrogenase (ADH) to acetaldehyde, which is then itself metabolized by aldehyde dehydrogenase (ALDH) to acetate. Acetate is then broken down into water and carbon dioxide for elimination. Acetaldehyde is rapidly metabolized by most individuals so that blood acetaldehyde levels typically remain low and it is unlikely to be present during AH. Nevertheless, acetaldehyde is highly toxic. It can cause tissue damage [20,21] and its presence in the body has been associated with hangover-like symptoms, including nausea, sweating, rapid pulse and headache [22,23]. It has been argued that increased acetaldehyde concentration and its long-lasting effects contribute to the presence of hangover symptoms [24,25]. However, the one human study to assess the effects of blood acetaldehyde levels on AH severity failed to find any evidence for a correlation between peak acetaldehyde concentration and hangover severity [26]. However, this one study does not provide sufficient evidence to exclude the possibility of an association between acetaldehyde and AH severity.

Evidence collected in several animal [27,28,29,30], human [31] and in vitro studies [32,33] indicate severe effects of ethanol on inflammatory processes. Inflammatory responses can also result in a variety of hangover-related symptoms, including nausea, vomiting, headache, negative mood and cognitive impairment [34,35]. Several studies have demonstrated evidence for elevated cytokine levels during hangover [31,36,37,38]. Another marker of inflammation, C-reactive protein (CRP), has been reported in two studies to correlate with AH severity [25,39], while another study has failed to find an association between CRP levels and AH [40]. However, the reliability of one of the studies that reported an association [25] is questionable as the assays that were used to measure CRP had limited detection sensitivity. This resulted in almost one-quarter of the data being outside the detection limit. Since this study, the development of highly sensitive assays to measure CRP levels have enabled more accurate measurement of CRP.

The current lack of understanding of the pathology of AH has hindered the development of an effective hangover treatment. Despite this there remains a high consumer demand [41] and many currently available products are advertised as mitigating AH severity. Yet there is no hangover treatment on the market with robust evidence for efficacy. Proposed treatments that have been investigated in human research showed either no effect, or minimal and differential reduction in the presence or severity of some but not other hangover symptoms [42].

The treatment of AH is further complicated by individual variation in hangover symptom frequency and severity [43,44], of which, genetic variations contribute about 40%–45% [45]. The influence of genetic variations on AH is evident when considering the efficacy of Korean pear juice, which has been shown to effectively reduce certain AH symptoms according to aldehyde dehydrogenase (ALDH) genotype. Specifically, it is effective in carriers of the ALDH2*1/*1 and ALDH2*1/*2 alleles, while being ineffective in the ALDH2*2/*2 genotype [46]. Variations in aldehyde dehydrogenase genes are also responsible for alcohol-induced flush reactions that are evident in 36% of people descended from East Asia [47]. Symptoms include flushing of the face, neck and shoulders, along with symptoms commonly associated with hangover, including headache and nausea, which are caused by elevated circulating levels of acetaldehyde [48]. Individuals who experience alcohol-induced flush reactions also display greater susceptibility to AH [49] and sensitivity to AH symptom severity [50], adding further support for the important role of acetaldehyde in AH. However, it should also be noted that Lee et al. [44] investigated the effects of Korean pear in a sample of 14 healthy male-only Asian subjects. Therefore, more research is needed to confirm these findings in groups of non-Asian descent men and women.

The aim of this investigation was to examine the effects of Rapid Recovery on AH symptom severity, inflammation, sleep quality and cognitive functioning. Rapid Recovery is an oral capsule that contains the amino acid L-cysteine and B and C group vitamins. It is proposed by the manufacturers that these ingredients will improve acetaldehyde metabolism and reduce oxidative stress. L-cysteine plays a role in reversing oxidization in the liver, with animal research showing that L-cysteine accelerates the breakdown and reduces the accumulation of acetaldehyde [51]. Another rodent study found that the administration of L-cysteine combined with vitamins B-1 and C reduced mortality caused by acetaldehyde poisoning [52]. In the current study, we tested the hypothesis that Rapid Recovery would reduce AH severity in social drinkers. A number of relevant biomarkers were co-monitored.

## 2. Experimental Section

### 2.1. Method

This study was conducted in accordance with the Declaration of Helsinki and was approved by the Swinburne University Human Research Ethics Committee (SUHREC, 2018/275). This study was registered with the Australian New Zealand Clinical Trials Registry (ANZCTR, ACTRN12618001996257).

### 2.2. Design

This study was a semi-naturalistic, randomized, double-blind, placebo-controlled, crossover clinical trial. The laboratory was set-up to simulate a bar-like environment and participants consumed alcoholic drinks of their choice and at their own pace, to a maximum of 1.3 g/kg alcohol. Participants were administered either placebo or active treatment over two testing visits.

### 2.3. Participants

Twenty-three participants who were healthy, aged 21–50 years old and regularly experienced hangovers were enrolled in the study. Three participants withdrew at the first morning visit, two withdrew due to illness and one failed to meet the eligibility requirement of a (BAC of 0.00% at the morning visit. The final sample consisted of 20 participants (65% female) with a mean age of 30.30 years (range 25–43 years old).

All participants were free of any current or history of drug or alcohol abuse, medically treated liver or renal impairment, pregnancy or breast feeding in females, and current use of any medication that could potentially affect the outcome of the study.

### 2.4. Measures

#### 2.4.1. Breath Alcohol Concentration (BAC)

BAC was measured at the beginning of each testing visit to ensure a reading of 0.00%. In the evening testing visits, BAC was measured approximately 20-min after the final alcoholic drink. BAC was collected using a regularly calibrated Lion Alcolmeter SD400PA.

#### 2.4.2. Assessment of Hangover Severity

Overall hangover severity was measured using a single one-item rating and severity of 23 hangover symptoms were rated on an 11-point Likert scale ranging from 0 to 10, with higher scores indicating a more severe hangover. The 23 items were derived from the Alcohol Hangover Severity Scale, the Hangover Symptoms Scale and the Acute Hangover Scale [53,54]. This composite scale has been successfully implemented in previous hangover research [55].

#### 2.4.3. Sleep Quality Assessments

Self-reported assessments of sleep quality comprised of the Groningen Sleep Quality Scale (GSQS) [56] and the Karolinska Sleepiness Scale (KSS) [57], which measured sleep quality during the previous night and current sleepiness, respectively. The GSQS comprises of 15 sleep complaints requiring a “yes” or “no” response indicating whether they had been experienced during the previous night’s sleep. Scores range from 0 to 14, (the first item is not scored) with higher scores indicating poorer sleep quality. The KSS requires participants to indicate their level of fatigue in the last five minutes on a single-item using a nine-point Likert scale. Higher scores indicate greater levels of sleepiness. These scales have been implemented successfully in previous hangover research [9,55].

#### 2.4.4. Assessment of Biomarkers for Inflammation and Liver Function

High-sensitivity C-reactive protein (hs-CRP) tests were used to measure inflammation and gamma-glutamyl transferase (GGT) tests were used to measure liver function.

#### 2.4.5. Assessment of Cognitive Performance

Cognitive performance was measured using the following tests available on the Vienna Test System (Schuhfried GmbH, Moedling, Austria). This test system assesses cognitive functioning that influence driving ability. The entire battery required approximately 15–20 min to complete.

##### Reaction Test (RT)

This test measures reaction time and motor time in response to optical and acoustic signals [58]. Participants were asked to place and leave their index finger on a pressure-sensitive key (i.e., rest key). Using the same index finger, participants were required to react as quickly as possible to the signals by pressing a target key before retiring their finger to the rest key. Performance was measured according to mean reaction time, mean motor time and number of correct reactions.

##### Determination Test (DT)

This test assesses reactive stress tolerance, divided attention and mental flexibility [59]. Participants are presented with various visual and auditory stimuli and are required to respond the stimuli by pressing corresponding response buttons with either their hands or feet, using the response panel and foot pedals of the Vienna Test System. Performance was assessed according to reaction time, number of correct responses, number of errors and number of missed responses.

##### Adaptive Tachistoscopic Traffic Perception Test (ATAVT)

This test assesses visual observation skills, visual orientation ability, speed of perception and skills in obtaining a traffic overview [60]. Images of traffic situations appeared briefly on a computer screen and the participant was asked to state what was in each image, by choosing from five answer options; motor vehicle, road sign, traffic light, pedestrian and bicycle. Performance was measured according to reaction time and the number of errors made.

#### 2.4.6. Perceived Treatment Order

Awareness of the allocated condition order (active-placebo or placebo-active) was measured at the end of the trial. Participants were asked which treatment (active or placebo) they believed they had received on the first and second testing visit.

### 2.5. Procedure

Prior to undergoing any testing procedures, participants provided written informed consent and were assessed for eligibility. Participants then underwent training and practice in completing the RT, DT and ATAVT tasks, and provided a baseline blood sample for hs-CRP and GGT analyses.

All testing visits were held in the laboratory, with intoxication visits held between 17:00 and 00:30, and hangover visits held the following morning between 7:00 and 11:00. The two evening visits were held within 7–14 days of one another. During the evening visits, the laboratory was set-up to mimic a bar and background music was played while participants socialized with one another.

Participants were advised to avoid alcohol for 24-h prior to the intoxication visits, food and drink (other than water) for 2-h prior to all testing visits and alcohol, drugs, food and caffeine between the evening and morning visits. At the beginning of each evening visit, participants were provided with a meal, the type and quantity of food consumed in the first visit was recorded and replicated in the second evening visit. Participants were then instructed to freely consume the drink type(s) of their choice (of wine, cider, beer, spirits), to a maximum of 1.3 g/kg alcohol. The time that each drink was started and finished was recorded and drinking behavior was replicated in the second evening visit. Participants were administered the first dose of the study treatment with their final drink and were provided the second dose to self-administer upon their first awakening the following morning. The study treatment was either placebo (corn flour) or Rapid Recovery (L-cysteine, thiamine, pyridoxine and ascorbic acid). The contents of the study treatments were controlled by a laboratory independent of the manufacturer.

Participants returned to the laboratory the following morning where they were initially breathalyzed to ensure a BAC reading of 0.00%. Once deemed eligible, participants were able to commence the testing procedures.

### 2.6. Statistics and Analyses

Statistical analyses were conducted using SPSS, Version 25 (IBM Corp, Armonk, NY, USA). All variables were analyzed using paired sample t-tests comparing Rapid Recovery with placebo. The sleep quality assessments and Hs-CRP and GGT levels were correlated with overall hangover severity. Lastly, a chi-square test was used to determine whether there was a significant difference between correct and incorrect perceived treatment order.

In order to further investigate whether the obtained data was more in favor of the null hypothesis (H0, i.e., the assumption of no differences between the active and placebo condition) or more in favor of the alternative hypothesis (H1, i.e., the assumption of differences between the active and placebo condition), add-on Bayesian statistics were conducted using the standard settings of SPSS for the respective tests. Based on the cutoffs suggested by Wagenmakers, et al. [61] the Bayes factor (BF) of 1 does not provide evidence for either hypothesis. Larger BF values provide stronger evidence for the H0 (compared to the H1), while smaller BF values provide stronger evidence for the H1 (compared to the H0), given the obtained data. Specifically, values 1–3 (1/3–1) are seen as anecdotal evidence for the H0, values 3–10 (1/10–1/3) are seen as substantial evidence for the H0, values of 10–30 (1/30–1/10) are seen as strong evidence for the H0, values of 30–100 (1/100–1/30) are seen as very strong evidence for the H0, and values of >100 (<1/100) are seen as extreme evidence for the H0.

## 3. Results

### 3.1. BAC Levels

BAC levels obtained in the active (mean = 0.096%, sd = 0.023) and placebo (mean = 0.097%, sd = 0.028) conditions did not significantly differ (*t*(19) = 0.507, *p* = 0.618). Add-on Bayesian analyses provided substantial evidence for the null hypothesis (BF = 5.183), showing that BAC concentrations did indeed not differ between conditions.

### 3.2. Hangover Symptom Severity

The only hangover symptom to significantly differ according to testing condition was “sleep problems” *t*(19) = 2.10, *p* = 0.049, with more severe sleep problems in the active (mean = 2.59, sd = 2.86) compared to placebo (mean = 1.63, sd = 1.75) condition. Following Bonferroni’s correction, this difference was no longer significant. Add-on Bayesian analyses for the non-significant effects revealed that in most cases, the obtained BF provided substantial evidence for the H0, as indicated by BF values between 3 and 10. For the other factors (“reduced appetite”, “sweating”, “heart beating” and “vomiting”), the Bayesian analyses still provided anecdotal evidence in favor of the H0, as indicated by BF values between 1 and 3. Taken together, all of these findings support the assumption that none of the investigated measures improved during the active condition. Hangover symptom severity scores can be found in Table 1, below.

### 3.3. Sleep Quality and Cognitive Performance

There were no significant differences between the Rapid Recovery and placebo conditions on the Groningen Sleep Quality Scale (GSQ), Karolinska Sleepiness Scale (KSS), reaction test (RT), determination test (DT) and adaptive tachistoscopic traffic perception test (ATAVT). The mean scores and standard deviations are displayed in Table 2, below. Self-rated overall hangover severity significantly correlated with GSQ (*r* = 0.552, *p* = 0.012) and KSS (*r* = 0.764, *p* ≤ 0.001) scores in the placebo condition. The same result was found in the treatment condition, with overall hangover severity scores significantly correlating with GSQ (*r* = 0.638, *p* = 0.002) and KSS (*r* = 0.762, *p* < 0.001) scores.

### 3.4. Levels of Biomarkers for Inflammation and Liver Function

There were no significant differences in hs-CRP and GGT levels in the Rapid Recovery compared to placebo condition (all *p* ≥ 0.376). Bayesian add-on analyses further provided substantial evidence for the H0 (all BF ≥ 3.587), thus demonstrating that both measures did not differ across conditions. Furthermore, hs-CRP and GGT levels did not significantly correlate with self-rated overall hangover severity in either of the testing conditions (all *p* ≥ 0.286). For both conditions, add-on Bayesian analyses provided substantial evidence for the lack of correlation between hs-CRP and overall hangover ratings (all BF ≥ 3.000) and for the lack of correlation between GGT and overall hangover (all BF ≥ 3.008). The hs-CRP and GGT levels can be found in Table 3, below.

### 3.5. Percieved Treatment Order and Adverse Events

A total of 60% of the participants guessed the correct condition order, indicating adequate blinding, χ^2^ = 0.80, *p* = 0.371.

There were no reported adverse events associated with Rapid Recovery.

## 4. Discussion

The current study assessed the effects of Rapid Recovery on hangover symptom severity. The hypothesis that Rapid Recovery would reduce AH severity was not supported. There were no significant differences between placebo and Rapid Recovery on self-rated overall hangover severity, sleep quality, CRP and GGT levels and cognitive performance. Furthermore, Bayesian add-on analyses provided credible evidence that the assumption of a null effect was more likely (than the assumption of non-significant/residual differences), given the obtained data. Of the 23 hangover symptoms that were assessed, the only significant difference between Rapid Recovery and placebo was found on the symptom of “sleep problems”, which was worse following Rapid Recovery administration. The ineffectiveness of Rapid Recovery to reduce AH severity may indicate that administration of l-cysteine combined with B and C vitamins does not improve acetaldehyde metabolism, or that acetaldehyde is not responsible for AH severity.

The results of this study further support the relationship between poor sleep quality and hangover severity [8,62,63,64], with significant and positive correlations between AH severity, and poor previous night’s sleep quality and current sleepiness. The current study failed to provide any evidence for a correlation between AH severity and CRP or GGT levels, which remained within normal ranges at each testing timepoint. Currently, the evidence for an association between AH severity and CRP is mixed, with some studies [25,39] demonstrating support for an association, while one other study [40], consistent with the findings of the current study, failed to find significant correlation between AH severity and CRP. While GGT is a reliable biomarker of liver damage caused by chronic heavy drinking [65,66,67], previous research has indicated that GGT levels are not associated with AH susceptibility [49] and, consistent with the findings of this study, are not necessarily elevated during AH [39]. Although it was not demonstrated in the current study, compelling evidence indicates an impairing effect of AH on immune functioning, and more reliable markers of this may include, but are not limited to, interleukin (IL)-6, IL-10, IL-12 and tumor necrosis factor (TNF)-α [37,68,69].

This investigation utilized a novel, controlled and ecologically valid methodology, which was found to successfully induce AH. Participants obtained mean BACs of 0.096% and 0.097% in the Rapid Recovery and placebo conditions, respectively, levels beyond that required to induce a hangover [70]. By enabling participants to self-administer alcohol within a controlled laboratory setting, we were able to overcome several commonly occurring methodological issues within the area of AH research. While methods of alcohol dosing used in previous laboratory studies assessing AH have been criticized for not mimicking real-life drinking behaviors, naturalistic studies have been criticized for lacking experimental control, and relying on self-reported alcohol intake to calculate estimated BAC [4,71,72,73]. The current study used methodology which combined the advantages of naturalistic approaches (i.e., participants drinking alcohol of their choice in a social setting) with those of laboratory studies (i.e., a controlled environment, objective measures of BAC and other biomarkers, veracity of treatment administration).

There were several limitations in this study. Firstly, we allowed participants to consume their preferred type of alcohol to ensure drinking behaviors replicated real-life drinking. Although drinking behaviors were consistent across the two testing visits, which eliminated intraindividual differences, this introduced interindividual variability. Alcoholic drinks contain various concentrations of congeners, which have been found to increase AH severity [17,74]. On the other hand, the fact that each individual’s session was matched, somewhat, mitigates against this influencing our results. Furthermore, it is possible that the effectiveness of Rapid Recovery is dependent on individual factors, for example, genetic variations or tolerance to alcohol. This was evident in literature on the effectiveness of Korean pear juice in treating certain AH symptoms in particular genetic subgroups but not others [46]. The sample size of this study was too small to allow meaningful subgroup analysis. Lastly, we did not assess hangover symptoms following alcohol abstinence because, although interesting, this was not necessary for the aim of this study, i.e., comparing Rapid Recovery and placebo. As such, we are unable to determine the severity of AH obtained in this study. While mean BAC levels are beyond those deemed required to induce a hangover [70], the mean overall hangover severity score is relatively low. Recent evidence indicates that BAC may not be the most appropriate predictor of AH severity, which is better predicted by levels of subjective intoxication and increased alcohol consumption compared to usual [70]. While these factors were not assessed in the current study, future AH research should aim to include measures of subjective intoxication and typical alcohol intake.

In conclusion, the findings from this study suggest that the administration of Rapid Recovery does not mitigate AH severity, and Hs-CRP and GGT levels are not associated with AH. Further research is required to assess the impact of an effective hangover treatment on alcohol consumption and determine whether it would encourage excessive drinking. Importantly, an effective hangover treatment would not mitigate all adverse factors associated with heavy drinking, such as chronic disease and injury. The development of an effective hangover treatment is currently hindered by a lack of understanding of the pathology of AH. As such, future research should continue to assess the pathology of AH to enable the development of treatments that target key mechanisms involved in the AH.

## Figures and Tables

**Table 1:** Hangover symptom severity scores (means and standard deviations) in the Rapid Recovery and placebo conditions. Descriptive data is given in the left columns, while the p value obtained from paired samples t-tests and the Bayes factor (BF) value obtained in case of non-significant differences (i.e., *p* values < 0.05) are provided in the right columns.

Item	Placebo M (SD)	Rapid Recovery M (SD)	*p* Value	BF Value
**Single-Item Severity Scale**				
‘How severe is your hangover?’	3.18 (2.69)	3.22 (2.07)	0.962	5.856
**Hangover Symptom Composite Scale**				
Concentration problems	5.65 (2.40)	5.43 (1.92)	0.748	5.570
Thirst	4.94 (2.51)	5.12 (1.35)	0.775	5.630
Tiredness	4.78 (2.35)	4.83 (2.43)	0.957	5.854
Sleepiness	4.53 (2.340	4.66 (2.50)	0.874	5.790
Headache	3.31 (3.07)	3.31 (2.51)	0.996	5.862
Apathy	2.90 (2.64)	2.79 (2.22)	0.856	5.768
Clumsiness	2.65 (2.01)	2.66 (2.11)	0.994	5.862
Weakness	2.54 (2.63)	2.71 (2.07)	0.835	5.737
Sensitivity to light	2.26 (2.54)	2.38 (2.06)	0.811	5.698
Nausea	1.77 (1.72)	2.37 (2.47)	0.365	3.904
Sleep problems	1.63 (1.75)	2.59 (2.86)	0.049 *	/
Reduced appetite	1.61 (1.80)	2.57 (2.75)	0.219	2.774
Dizziness	1.53 (1.54)	2.05 (1.97)	0.360	3.870
Stomach pain	1.37 (2.38)	1.35 (2.08)	0.977	5.860
Shaking, shivering	1.23 (1.66)	0.95 (1.31)	0.407	4.169
Anxiety	1.18 (1.54)	1.07 (1.20)	0.668	5.350
Confusion	1.17 (1.54)	0.98 (0.95)	0.569	4.991
Regret	1.05 (1.68)	0.77 (1.00)	0.429	4.298
Sweating	0.93 (1.08)	1.40 (1.68)	0.238	2.940
Heart beating	0.90 (1.31)	1.41 (1.76)	0.234	2.903
Depression	0.73 (0.88)	0.75 (0.97)	0.935	5.843
Heart racing	0.67 (0.95)	0.84 (0.93)	0.247	3.016
Vomiting	0.39 (0.53)	0.89 (1.70)	0.193	2.529

Note: M: Mean; SD: Standard deviation; *: *p* < 0.05.

**Table 2:** Sleep quality and cognitive performance scores (means and standard deviations) in the Rapid Recovery and placebo conditions. Descriptive data is given in the left columns, including Groningen Sleep Quality Scale (GSQS), Karolinska Sleepiness Scale (KSS), reaction test (RT), determination test (DT) and adaptive tachistoscopic traffic perception test (ATAVT) while the *p* value obtained from paired samples t-tests and the BF value obtained in case of non-significant differences (i.e., *p* values < 0.05) are provided in the right columns.

Item	Rapid Recovery M (SD)	Placebo M (SD)	*p*-Value	BF Value
**Sleep Quality**				
GSQ	4.10 (3.54)	3.35 (3.01)	0.429	4.300
KSS	5.00 (2.15)	4.85 (2.06)	0.845	5.753
**RT**				
Reaction time (milliseconds)	426.79 (57.23)	415.37 (79.40)	0.297	3.341
Motor time (milliseconds)	152.84 (37.55)	156.89 (34.64)	0.584	4.788
Number of correct reactions	15.95 (0.23)	16.00 (0.00)	0.331	3.580
**DT**				
Reaction time (milliseconds)	676.80 (61.20)	668.90 (62.35)	0.544	4.770
Number of correct responses	283.05 (33.58)	289.32 (27.03)	0.367	3.826
Number of errors	21.42 (11.76)	22.21 (11.54)	0.681	5.265
Number of missed responses	14.21 (8.34)	13.95 (6.93)	0.823	5.586
**ATAVT**				
Reaction time (seconds)	8.98 (1.50)	9.03 (1.47)	0.893	5.675
Number of errors	5.95 (2.37)	6.00 (3.79)	0.956	5.718

Note: M: Mean; SD: Standard deviation.

**Table 3:** High sensitivity C-reactive protein (hs-CRP) and gamma-glutamyl transpeptidase (GGT) levels (means and standard deviations) at baseline and in the Rapid Recovery and placebo conditions. Descriptive data is given in the left columns, while the *p* value obtained from paired samples t-tests comparing the placebo and active condition, as well as the BF value obtained in case of non-significant differences (i.e., *p* values < 0.05) are provided in the right columns (*N* = 16).

	Baseline M (SD)	Rapid Recovery M (SD)	Placebo M (SD)	*p*-Value	BF Value
Hs-CRP (mg/L)	1.78 (2.86)	1.49 (2.28)	1.43 (2.37)	0.813	5.150
GGT (U/L)	27.56 (14.39)	28.31 (15.17)	27.13 (13.85)	0.376	3.587

Note: M: Mean; SD: Standard deviation.
